# Enhancing Preoperative Diagnostic Accuracy in Endometrial Hyperplasia: A Comparison of Biopsy Methods

**DOI:** 10.7759/cureus.81880

**Published:** 2025-04-08

**Authors:** Keita Asano, Hikari Unno, Satoshi Kubota, Satoko Matsuzaki, Tomoko Sumikura, Tadashi Iwamiya, Kohki Shimazu, Hiroaki Fushimi, Masahiko Takemura, Ken-ichiro Morishige

**Affiliations:** 1 Department of Obstetrics and Gynecology, Osaka University, Osaka, JPN; 2 Department of Obstetrics and Gynecology, Osaka General Medical Center, Osaka, JPN; 3 Department of Pathology, Osaka General Medical Center, Osaka, JPN

**Keywords:** dilatation and curettage (d&c), endometrial aspiration biopsy, endometrial cancer, endometrial hyperplasia, endometrial intraepithelial neoplasia (ein)

## Abstract

Introduction

This study aimed to evaluate the diagnostic accuracy of aspiration biopsy alone versus a combined aspiration biopsy and full curettage for endometrial hyperplasia, focusing on concerns about the underestimation of malignancy with aspiration alone.

Methods

We compared the pathological diagnoses obtained from aspiration biopsy and full curettage with postoperative diagnoses in 87 surgically treated cases of endometrial hyperplasia at our center (August 2013 to September 2023). The diagnostic accuracy of each preoperative histology method was compared against the final postoperative diagnosis for cases where intraoperative rapid pathology was performed, either with aspiration biopsy alone or with combined aspiration and curettage.

Results

Aspiration biopsy alone diagnosed 47 cases (54.0%) as endometrial intraepithelial neoplasia (EIN) and 40 cases (46.0%) as endometrial hyperplasia without atypia. Preoperative histological diagnosis was performed by aspiration alone in 63 cases (72.4%) and by aspiration plus full curettage in 24 cases (27.6%). The rate of postoperative upgrade to endometrial cancer was significantly lower when both methods were used (42.9% vs. 16.7%; p = 0.026, Fisher’s exact test). Among 33 cases with intraoperative rapid pathology, the upgrade rate was lower in the combined method group than in the aspiration-only group, though the difference was not statistically significant (30.8% vs. 14.3%; p = 0.64).

Conclusions

Incorporating full curettage alongside aspiration biopsy improves diagnostic accuracy for endometrial hyperplasia, reducing the risk of misdiagnosis and aiding appropriate treatment decisions. These results emphasize the importance of considering both biopsy methods in preoperative diagnosis and management.

## Introduction

Endometrial hyperplasia is a pathological condition marked by the atypical proliferation of the endometrial glands and stroma. Particularly, endometrial hyperplasia with atypia is considered a precursor lesion to endometrial cancer and has significant clinical relevance. Accurate diagnosis of pre-malignant lesions and the exclusion of coexisting endometrial carcinoma are crucial for the management of endometrial hyperplasia.

The classification system established by the World Health Organization (WHO) in 1994 categorizes endometrial hyperplasia into four distinct types, which are determined by the degree of glandular complexity and the presence of cytological nuclear atypia: simple hyperplasia without atypia, complex hyperplasia without atypia, simple atypical hyperplasia, and complex atypical hyperplasia [1]. However, the American College of Obstetricians and Gynecologists and the Society of Gynecologic Oncology now recommend using the endometrial intraepithelial neoplasia (EIN) classification [2]. The EIN classification divides endometrial hyperplasia into three categories: benign (benign endometrial hyperplasia), precancerous (EIN), and malignant (endometrial cancer). EIN is considered a more accurate indicator of future cancer risk than the WHO 1994 classification, and approximately 30% of EIN cases progress to endometrial cancer [3].

Various techniques for endometrial sampling are employed to diagnose endometrial lesions, including endometrial hyperplasia. Dilation and curettage (D&C) has long been a widely used method, though it is sometimes not performed due to the risks associated with anesthesia and complications. Endometrial aspiration biopsy has largely replaced D&C due to its ease, safety, and simplicity [4,5]. However, limited reliable data is comparing the diagnostic accuracy of D&C versus aspiration biopsy for endometrial lesions [6], and studies with large sample sizes specifically for endometrial hyperplasia are lacking.

Some studies comparing the diagnostic accuracy of D&C and aspiration biopsy for endometrial hyperplasia used the WHO 1994 classification [7]. However, no studies have evaluated these methods using the EIN classification, which is currently the gold standard. Therefore, this study aimed to compare the diagnostic accuracy of preoperative aspiration biopsy alone versus a combination of aspiration biopsy and D&C in patients diagnosed with endometrial hyperplasia using the EIN classification before hysterectomy.

In clinical practice, frozen section pathology is often used during hysterectomy to accurately assess endometrial cancer when a preoperative diagnosis of atypical endometrial hyperplasia is made. Thus, we also compared diagnostic accuracy between aspiration biopsy and D&C in surgeries involving intraoperative rapid pathology.

## Materials and methods

This study was approved by the Ethical Review Committee of Osaka General Medical Center (approval number: 2024-005) and carried out in compliance with the ethical guidelines established by the Declaration of Helsinki. The requirement for informed consent was dispensed with. We conducted a retrospective review of patients who underwent surgery between August 2013 and September 2023 at Osaka General Medical Center, a tertiary hospital in Japan, based on electronic medical record data.

We examined the medical records of 87 patients diagnosed with endometrial hyperplasia through aspiration biopsy or D&C who subsequently underwent hysterectomy. Aspiration biopsies were performed without anesthesia using a Pipelle device (Pipet Curet™ Cooper Surgical Inc., USA), while D&C was performed under sedation with midazolam and pentazocine.

Eligible patients were those diagnosed with endometrial hyperplasia by aspiration biopsy, including EIN and endometrial hyperplasia without atypia. Pathologists reclassified EIN cases using the EIN classification since cases before 2015 were diagnosed according to the WHO 1994 criteria. Patients diagnosed with endometrial cancer by aspiration biopsy or with insufficient tissue for pathological evaluation were excluded.

Pathological diagnosis by D&C was performed within three months of the aspiration biopsy. For intraoperative rapid pathology, a frozen section of the tumor lesion was prepared immediately after hysterectomy and examined by a pathologist for intraoperative diagnosis. The choice of diagnostic method (aspiration biopsy alone, aspiration biopsy with D&C, or intraoperative rapid pathology) was left to the attending physician’s discretion. Hysterectomy was performed for preoperative diagnoses of EIN or higher. Surgery was also considered for cases of endometrial hyperplasia without atypia if persistent irregular bleeding, uterine myomas, uterine prolapse, or other comorbidities were present, or if the patient strongly desired the procedure.

All surgeries were performed by gynecologic oncologists, and no patients received progesterone therapy prior to surgery. The preoperative endometrial histology results were compared with the diagnosis from the excised uterine specimen. We also evaluated the consistency of pathological results between aspiration biopsy and D&C specimens. Results of “no residual lesions,” “secretory stage endometrium,” and “proliferative stage endometrium” in postoperative specimens were considered normal. Fisher's exact test was used to compare categorical variables, and a p-value of <0.05 was considered statistically significant.

## Results

A total of 87 patients were included in this study. Patient demographics are shown in Table 1. Of the 87 patients, 63 underwent aspiration biopsy alone and 24 underwent both aspiration biopsy and D&C. The mean age was 54.1 (±11.5) years, and the mean BMI was 25.5 (±6.5).

**Table 1:** Characteristic of patients

Characteristics of the patients	Values
Age, years, Mean±SD	54.1±11.5
Body mass index (BMI), kg/m^2^	25.5±6.5
CA125, U/mL	22.9±75.7
CA19-9, U/mL	23.6±42.5
Method of biopsy, n (%)	
Aspiration biopsy only	63 (72.4)
Aspiration biopsy＋D&C (dilation and curettage)	24 (27.6)
Endometrial sampling pathology by aspiration biopsy	
Endometrial hyperplasia (EH) without atypia	40 (46.0)
Atypical endometrial intraepithelial neoplasia (EH/EIN)	47 (54.0)
Endometrial sampling pathology by D&C	
Normal	2 (8.4)
EH without atypia	6 (25.0)
Atypical EH/EIN	11 (45.8)
Carcinoma	5 (20.8)
Hysterectomy pathology	
Normal	22 (25.3)
EH without atypia	12 (13.8)
Atypical EH/EIN	17 (19.6)
Endometrioid carcinoma	34 (39.1)
Serous carcinoma	1 (1.1)
Mucinous carcinoma	1 (1.1)
Surgical procedure	
Laparoscopic	41 (47.1)
Robotic	11 (12.6)
Abdominal	32 (36.8)
Vaginal	3 (3.5)

Histological results from aspiration biopsy revealed endometrial hyperplasia without atypia in 40 cases (46.0%) and EIN in 47 cases (54.0%). Histological results from D&C showed normal endometrium in two cases (8.4%), endometrial hyperplasia without atypia in six cases (25.0%), EIN in 11 cases (45.8%), and cancer in five cases (20.8%). The final pathological diagnosis after hysterectomy was normal endometrium in 22 patients (25.3%), endometrial hyperplasia without atypia in 12 patients (13.8%), EIN in 17 patients (19.6%), and cancer in 36 patients (41.3%). The postoperative stage (Federation of Gynecologists and Obstetricians (FIGO) 2008) was stage IA in 29 cases (80.6%) and stage IB in nine cases (19.4%). Forty-one patients (47.1%) underwent laparoscopic surgery, 11 (12.6%) had robotic-assisted surgery, 32 (36.8%) underwent open surgery, and three (3.4%) had vaginal surgery.

Comparing diagnoses between aspiration biopsy and hysterectomy specimens, 36 patients (41.4%) had their diagnosis upgraded to cancer postoperatively (Table 2). Of the 40 patients diagnosed with endometrial hyperplasia without atypia, 5 (12.5%) were upgraded to cancer. Of the 47 patients diagnosed with EIN, 31 (65.9%) were upgraded.

**Table 2:** Upgrading risks to carcinoma with aspiration biopsy EH, Endometrial hyperplasia; EIN, Endometrial intraepithelial neoplasia

Aspiration biopsy	n (%)	Hysterectomy				Upgrade from biopsy, n (%)
		Normal	EH without atypia	Atypical EH/EIN	Carcinoma	
EH without atypia	40 (46.0)	18 (45.0%)	12 (30.0%)	5 (12.5%)	5 (12.5%)	5 (12.5)
Atypical EH/EIN	47 (54.0)	4 (8.5%)	0 (0%)	12 (25.5%)	31 (66.0%)	31 (65.9)
Total	87	22 (25.3%)	12 (13.8%)	17 (19.5%)	36 (41.4%)	36 (41.4)

Next, we compared the proportion of patients upgrading to carcinoma in the aspiration biopsy alone group and the combined aspiration biopsy and D&C group (Table 3). If the diagnoses from aspiration biopsy and D&C differed, the more advanced diagnosis was adopted. Among 24 patients in the combined biopsy group, four (16.7%) had their diagnosis upgraded to cancer postoperatively. The rate of upgrade to carcinoma was significantly lower in the combined D&C group compared to the aspiration biopsy alone group (16.7% vs. 42.9%; p = 0.026).

**Table 3:** Comparison of pathological upgrade to carcinoma risks of aspiration biopsy only and aspiration biopsy plus D&C P-value for Fisher's exact test (levels of significance: 0.05*) EM, Endometrial; EH, Endometrial hyperplasia; EIN, Endometrial intraepithelial neoplasia

Preoperative pathology	Upgrade to carcinoma on final pathology	EM sampling method		P-value
		Aspiration biopsy only	Aspiration biopsy＋D&C	
EH without atypia	No	25 (92.6%)	8 (100%)	
	Yes	2 (7.4%)	0	
Atypical EH/EIN	No	11 (30.6%)	7 (63.6%)	0.138*
	Yes	25 (69.4%)	4 (36.4%)	
Carcinoma	No	0	5 (100%)	
	Yes	0	0	
Total	No	36 (57.1%)	20 (83.3%)	0.026*
	Yes	27 (42.9%)	4 (16.7%)	

For the 33 patients who underwent intraoperative rapid pathology, the upgrade rate to cancer was lower in the combined D&C group (14.3%) compared to the aspiration-only group (30.8%), although this difference was not statistically significant (p = 0.64) (Table 4). Four patients (two in each group) required additional surgery after the pathology diagnosis was upgraded to cancer, including bilateral adnexectomy in three cases and bilateral adnexectomy with pelvic lymph node dissection in one. Six patients (6.9%) required chemotherapy postoperatively, with regimens including TC (paclitaxel plus carboplatin), DC (docetaxel plus carboplatin), and TC followed by Lenvatinib plus pembrolizumab. One patient (1.1%) died due to sepsis unrelated to gynecological surgery.

**Table 4:** Comparison of pathological upgrade to carcinoma risks in a case with intraoperative rapid pathology P-value for Fisher's exact test (levels of significance: 0.05*) EM, Endometrial; EH, Endometrial hyperplasia; EIN, Endometrioid intraepithelial neoplasia

Diagnosis based on preoperative plus intraoperative pathology	Upgrade to carcinoma on final pathology	EM sampling method		P-value
		Aspiration biopsy only	Aspiration biopsy+D&C	
EH without atypia	No	6 (100%)	1 (100%)	
	Yes	0	0	
Atypical EH/EIN	No	5 (38.5%)	1 (50.0%)	
	Yes	8 (61.5%)	1 (50.0%)	
Carcinoma	No	7 (100%)	4 (100%)	
	Yes	0	0	
Total	No	18 (69.2%)	6 (85.7%)	0.64
	Yes	8 (30.8%)	1 (14.3%)	

## Discussion

Our comparison of aspiration biopsy alone versus aspiration biopsy combined with D&C for diagnosing preoperative endometrial hyperplasia suggests that D&C combined with aspiration biopsy is a more accurate sampling method. In addition, the diagnostic accuracy may improve further when intraoperative rapid pathology is included. Similar findings were reported in a previous study comparing D&C and frozen section pathology for endometrial hyperplasia [8,9].

Patients diagnosed with endometrial hyperplasia face the possibility of undetected cancer and the potential development of endometrial carcinoma. For women with endometrial hyperplasia that does not exhibit atypia, the risk of progression is under 5%, whereas it increases to 30% for those with endometrial intraepithelial neoplasia (EIN) [3]. Therefore, hysterectomy is often recommended for patients diagnosed with EIN. However, for women who wish to preserve fertility or who cannot have surgery because of medical complications, conservative management is the only option. In these cases, accurate preoperative diagnostic evaluation is crucial, as the final pathological diagnosis cannot be confirmed until surgery.

A previous single-center study from Korea [7] evaluated the diagnostic accuracy of aspiration biopsy versus D&C in 250 cases of endometrial hyperplasia. The study found that D&C had significantly better diagnostic concordance with final pathology and a lower rate of postoperative upgrade to endometrial cancer. Furthermore, a prospective multicenter study demonstrated that D&C was more accurate than aspiration biopsy for follow-up in patients treated with progestin for endometrial hyperplasia [10,11]. However, these studies used the older WHO 1994 classification for diagnosing endometrial hyperplasia. There are several advantages in our study: it uses the currently accepted EIN classification for endometrial hyperplasia, and it also examines diagnostic accuracy in cases where intraoperative rapid pathology was performed. Additionally, we explored the consequences of inaccurate preoperative diagnosis, specifically its impact on patients who required reoperation or postoperative chemotherapy.

There are also studies suggesting that hysteroscopic biopsy and manual vacuum aspiration (MVA) systems are useful as endometrial biopsy methods. In the case of hysteroscopic biopsy, there have been reports examining the accuracy of the grade and histological type of endometrial cancer, but there have been no reports examining the accuracy of the diagnosis of endometrial hyperplasia [12,13]. Even in the case of the MVA system, studies on the accuracy of the diagnosis of endometrial hyperplasia are based on a small number of cases and are insufficient as evidence [14]. Therefore, whether hysteroscopic biopsy or MVA can replace D&C as a biopsy method for endometrial hyperplasia remains a subject for future research.

Limitations

One limitation of this study is the relatively small sample size due to its single-center design. In particular, the number of cases with intraoperative rapid pathology was small, limiting our ability to perform a robust statistical analysis for this subgroup. Additionally, the choice of biopsy method, whether aspiration biopsy alone or aspiration biopsy combined with D&C, was determined by the attending physician, which may have introduced selection bias. For example, patients with symptoms like frequent genital bleeding or abnormal thickening of the endometrium on transvaginal ultrasound may have been more likely to receive a combined biopsy approach. This could have resulted in a higher detection rate of atypical hyperplasia, EIN, or carcinoma in this group.

## Conclusions

Combined D&C is superior to aspiration biopsy alone for accurate preoperative diagnosis of endometrial hyperplasia. The risk of upgrading to endometrial cancer is higher with EIN than with endometrial hyperplasia without atypia. In practice, when endometrial hyperplasia is diagnosed by outpatient aspiration biopsy, additional D&C should be considered for reevaluation. However, even with optimal preoperative testing and intraoperative rapid pathology, there remains a risk of postoperative upgrading to cancer. Physicians should discuss the possibility of reoperation or chemotherapy with patients before surgery.
